# Influence of moderate left subclavian artery stenosis on outcomes after coronary bypass surgery in patients requiring hemodialysis

**DOI:** 10.1016/j.xjon.2026.101679

**Published:** 2026-02-12

**Authors:** Ryoma Oda, Takeshi Kinoshita, Daisuke Endo, Kan Kajimoto, Taira Yamamoto, Atsushi Amano, Minoru Tabata

**Affiliations:** a Department of Cardiovascular Surgery, Juntendo University School of Medicine, Tokyo, Japan; b Department of Cardiovascular Surgery, Juntendo University School of Medicine, Shizuoka Hospital, Shizuoka, Japan; c Department of Cardiovascular Surgery, Juntendo University School of Medicine, Nerima Hospital, Tokyo, Japan

**Keywords:** coronary artery bypass grafting, coronary steal syndrome, hemodialysis patients, long-term outcomes, subclavian artery stenosis

## Abstract

**Objectives:**

Left subclavian artery stenosis in patients requiring hemodialysis can cause coronary steal syndrome via the left internal thoracic artery (LITA) graft to the left anterior descending artery (LAD), potentially leading to graft failure. This study evaluated the influence of moderate left subclavian artery stenosis on long-term outcomes following LITA-LAD coronary artery bypass grafting.

**Methods:**

Among 1744 patients undergoing primary isolated coronary artery bypass grafting, 104 patients requiring hemodialysis with left upper limb arteriovenous fistulas and preoperative contrast-enhanced computed tomography were analyzed. Left subclavian artery stenosis was quantified as: (1–minimal lumen area/reference lumen area) × 100. Patients were grouped by stenosis severity: ≥50% (n = 25) versus <50% (n = 79). The primary end point was all-cause mortality; secondary end points included cardiac death and major adverse cardiac events (eg, heart failure admission, ischemic events, or cardiac death). Inverse probability of treatment weighting was used for covariate adjustment, and inverse probability of treatment weighting-adjusted Cox models estimated hazard ratios.

**Results:**

Mean follow-up was 4.6 ± 3.1 years. In the inverse probability of treatment weighting cohort, 5-year survival was 62% versus 26% (nonstenosis vs stenosis; log-rank *P* < .001). Left subclavian artery stenosis was associated with higher risks of all-cause death (hazard ratio, 2.09; 95% CI, 1.39-3.15; *P* < .001) and cardiac death (hazard ratio, 3.68; 1.46-9.30; *P* = .006), but not with major adverse cardiac events.

**Conclusions:**

Moderate preoperative left subclavian artery stenosis was independently associated with increased long-term mortality and cardiac death after LITA-LAD coronary artery bypass graft in patients requiring hemodialysis. Routine preoperative subclavian imaging and consideration of revascularization or alternative inflow may be warranted.

## Graphical Abstract

**Figure:**
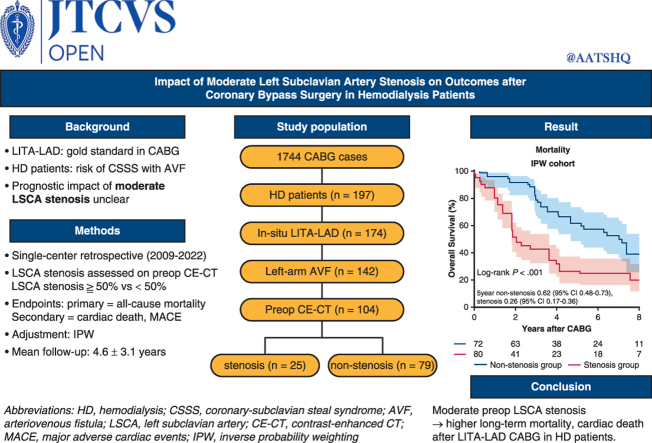

**Figure:**
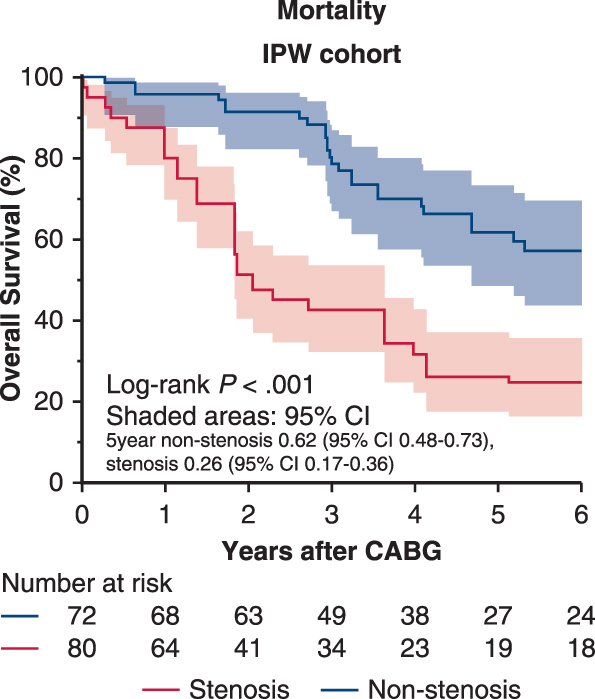
Moderate LSCA stenosis worsens survival after LITA-LAD CABG in hemodialysis (95% CI).

**Central Message**

In patients requiring hemodialysis with LITA-LAD CABG, moderate LSCA stenosis is associated with worse survival and cardiac death, supporting subclavian scans and tailored graft planning.

**Perspective**

Although in situ LITA grafting ipsilateral to an AVF is common, LSCA stenosis raises concern for CSSS and its outcome influence has not been well studied. In our cohort, ≥50% LSCA stenosis was linked to worse long-term survival and more cardiac death. These findings support routine preoperative subclavian imaging and adjustment of graft design.

The left internal thoracic artery (LITA) to left anterior descending artery (LAD) graft remains the cornerstone of surgical revascularization, offering unmatched long-term patency.^1^^,^^2^ However, in patients requiring hemodialysis, unique hemodynamics can challenge this paradigm. The presence of a left arteriovenous fistula (AVF) and concomitant subclavian artery (SCA) stenosis may divert blood flow from the LITA graft toward the upper limb—a phenomenon known as coronary-subclavian steal (CSS)—compromising myocardial perfusion.^3^, ^4^, ^5^

CSS syndrome (CSSS) is often underdiagnosed because its manifestations, such as exertional angina or heart failure, are nonspecific. Nevertheless, it carries serious prognostic implications; a recent meta-analysis reported that combined symptomatic and asymptomatic CSSS affects more than 10% of patients receiving dialysis with AVFs,^4^ suggesting the condition is substantially underdiagnosed. Although some studies suggest that ipsilateral LITA grafting is safe,^6^, ^7^, ^8^, ^9^, ^10^ others have documented dialysis-induced ischemia.^11^^,^^12^ This inconsistency indicates that AVF laterality alone is an insufficient predictor of risk and that the integrity of the subclavian inflow may be the decisive factor.^13^^,^^14^

Severe left SCA (LSCA) stenosis is a recognized contraindication to in situ LITA use. However, the clinical significance of moderate lesions—common yet often overlooked—remains uncertain in patients requiring hemodialysis. Current evidence is limited to small series, leaving their influence on long-term survival unanswered.

Accordingly, this study aimed to determine whether moderate preoperative LSCA stenosis is independently associated with increased long-term all-cause mortality, cardiac death, and major adverse cardiac events (MACE) in patients requiring hemodialysis undergoing coronary artery bypass grafting (CABG) with a LITA-LAD graft. Clarifying this association may refine preoperative risk stratification, guide inflow management—including consideration of free LITA grafting or preoperative subclavian intervention—and emphasize the necessity of routine SCA evaluation in this high-risk population.

## Materials and Methods

### Ethics and Design

**Figure 1:**
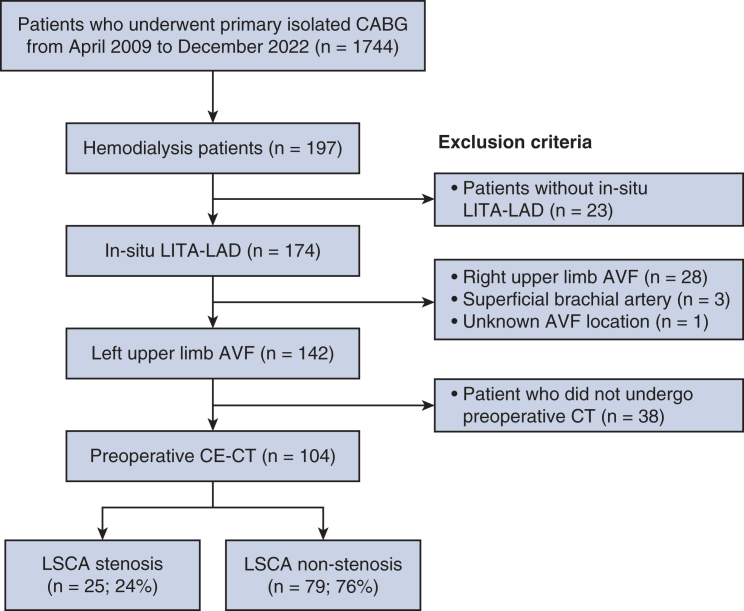
Patient selection flow diagram. Patients receiving hemodialysis undergoing coronary artery bypass grafting (CABG) with in-situ left internal thoracic artery-to-left anterior descending (LITA-LAD) and a left-arm arteriovenous fistula (AVF) were identified; exclusions and grouping by left subclavian artery (LSCA) stenosis are shown (n = 104). *CE-CT*, Contrast-enhanced computed tomography.

This retrospective study was approved by the Juntendo University Research Ethics Committee (E24-0018-H01; March 22, 2024), with informed consent waived owing to the retrospective design. All procedures complied with the Declaration of Helsinki. We reviewed 1744 patients undergoing primary isolated CABG at our institution between April 2009 and December 2022. Inclusion criteria were maintenance hemodialysis with a left-arm AVF, LITA-LAD anastomosis, and preoperative contrast-enhanced computed tomography (CT). Exclusion criteria included right-sided/unidentified AVFs or no preoperative CT (Figure 1). Clinical data followed Society of Thoracic Surgeons definitions.

### Operative Procedure

Procedures were performed via median sternotomy, with off-pump CABG as the primary strategy. Arterial grafts were skeletonized using a Harmonic scalpel (Ethicon). Saphenous vein grafts were harvested using the open technique; endoscopic harvesting was rare, and the nontouch technique was not used. The in situ right gastroepiploic artery (GEA) was routed transdiaphragmatically to revascularize the distal right coronary territory. The GEA was preferred for chronic total occlusions or severe stenosis (>90%) of the right coronary artery to avoid competitive flow. An aortic no-touch strategy was adopted for significant aortic plaque or calcification. Intraoperative graft flow was routinely assessed using transit-time flow measurement (VeriQ; Medistim ASA) when systolic blood pressure exceeded 100 mm Hg. Postoperatively, aspirin, statins, and beta-blockers were standard unless contraindicated.

### Outcomes

The primary end point was all-cause mortality. Secondary end points were cardiac death and MACE (composite of heart failure admission, ischemic events [nonfatal myocardial infarction or clinically indicated revascularization], and cardiac death). Cardiac death included death from heart failure, myocardial infarction, lethal arrhythmia, or sudden unexpected death within 24 hours of symptom onset. Events were adjudicated via medical records, dialysis clinics, and family interviews; causes that could not be determined were classified as undetermined (detailed in Table E1). Nonroutine late graft patency (CT or angiography) is summarized in Table E2.

### Follow-up

Patients underwent regular clinical assessments, including chest radiography, electrocardiography, and echocardiography. Clinical events and readmissions were captured through structured questionnaires. For patients missing hospital visits, follow-up was conducted via telephone interviews with patients or their dialysis clinics.

### Evaluation of Stenosis

Preoperative contrast-enhanced CT was used to assess LSCA stenosis. Orthogonal cross-sectional images were generated using a dedicated workstation (ZIOstation2; Ziosoft). The minimal lumen area at maximal narrowing and reference areas from adjacent normal segments were manually traced, and stenosis severity was expressed as percent area stenosis: (1−minimal lumen area/reference lumen area) × 100.

### Statistical Analysis

Continuous variables were compared using Student *t*-test or Mann-Whitney *U* test, and categorical variables with the χ^2^ test. Survival and cardiac death were estimated using the Kaplan-Meier method. MACE was analyzed as a time-to-first-event end point, with Gray test and Fine-Gray models accounting for the competing risk of noncardiac death.

To reduce treatment selection bias, inverse probability of treatment weighting (IPTW) was performed. Propensity scores were derived via logistic regression using 17 covariates (indicated in Table 1). Patients with extreme propensity score (<0.10 or >0.90) were trimmed. Covariate balance was assessed using standardized mean differences (SMD), with ∣SMD∣≤0.10 indicating adequate balance.^15^ As a doubly robust sensitivity analysis (Table E3), we additionally adjusted for covariates with meaningful residual imbalance after IPTW (SMD ≥0.20). Reproducibility of LSCA measurements was tested in 20 random cases using intraclass correlation coefficients for inter- and intraobserver reliability.^16^ Analyses were performed with SPSS 25.0 (IBM) and R (R Foundation for Statistical Computing).

**Table 1:** Patient characteristics

Variables	Original cohort (n = 104)				IPTW-weighted cohort (n = 154.6)		
	Stenosis (n = 25)	Non-stenosis (n = 79)	*P* value	SMD	Stenosis (n = 79.4)	Nonstenosis (n = 75.2)	SMD
Age (y)∗	67 ± 8.3	63 ± 10	.061	0.46	66 ± 9.0	66 ± 8.3	<0.01
≥70 y	10 (40)	22 (28)	.321	0.26	33 (41)	28 (37)	0.08
Male sex∗	21 (84)	68 (86)	.753	0.06	65 (82)	63 (84)	0.05
Body mass index∗	24 ± 5.5	24 ± 3.9	.602	0.11	24 ± 5.1	24 ± 4.1	0.03
Hypertension∗	21 (84)	64 (81)	>.99	0.08	68 (85)	65 (86)	0.01
Smoking history∗	19 (76)	47 (59)	.159	0.36	56 (71)	56 (75)	0.08
Cerebrovascular disease∗	7 (28)	13 (16)	.246	0.28	14 (18)	15 (20)	0.06
Peripheral artery disease∗	15 (60)	33 (42)	.167	0.37	47 (59)	43 (57)	0.04
COPD∗	1 (4)	2 (2.5)	.566	0.08	2 (2.5)	3 (4.0)	0.08
Diabetes mellitus	20 (80)	57 (72)	.602	0.18	62 (78)	64 (85)	0.20
Insulin∗	9 (36)	25 (32)	.807	0.09	30 (38)	28 (37)	0.02
†Primary cause of ESRD: Diabetes∗	20 (80)	53 (67)	.316	0.30	62 (78)	60 (80)	0.06
Dialysis duration (y)∗	5.9 (1.5-9.2)	5.8 (2.9-9.4)	.828	0.05	4.9 (0.8-9.4)	5.2 (2.2-8.6)	0.08
AVF, forearm∗	24 (96)	71 (90)	.684	0.24	76 (95)	71 (95)	0.02
AVF, graft	0 (0)	3 (3.8)	>.99	0.28	0 (0)	2 (2.7)	0.23
LDL-C (mg/dL)	77 ± 26	78 ± 26	.892	0.03	77 ± 22	77 ± 28	0.01
LVDd (mm)	53 ± 5.7	53 ± 6.6	.937	0.02	52 ± 5.3	53 ± 6.5	0.14
LVDs (mm)	39 ± 7.8	39 ± 8.7	.772	0.07	39 ± 7.2	39 ± 8.4	0.07
LVEF (%)∗	49 ± 14	50 ± 14	.849	0.04	50 ± 13	49 ± 13	0.05
LVEF <40%	7 (28)	21 (27)	>.99	0.03	18 (23)	20 (27)	0.09
No of diseased vessels∗	3.2 ± 0.9	3.1 ± 0.8	.437	0.17	3.2 ± 0.9	3.2 ± 1.0	0.01
1 or 2	5 (20)	15 (19)	>.99	0.03	18 (24)	23 (21)	0.06
3	12 (48)	44 (56)	.646	0.15	36 (46)	34 (45)	0.02
4 or 5	8 (32)	20 (25)	.606	0.15	24 (30)	26 (34)	0.09
LMT ≥50%∗	10 (40)	20 (25)	.206	0.32	29 (37)	27 (36)	0.02
LAD ≥75%	24 (96)	77 (97)	.566	0.08	76 (95)	72 (96)	0.05
LAD CTO	2 (8.0)	4 (5.1)	.629	0.12	6 (7.6)	5 (6.7)	0.04
RCA lesion	22 (88)	63 (80)	.553	0.23	67 (85)	57 (76)	0.22
RCA CTO	9 (36)	18 (23)	.200	0.29	28 (35)	15 (20)	0.35
LCx lesion	18 (72)	64 (81)	.401	0.21	59 (75)	64 (85)	0.27
LCx CTO	5 (20)	7 (8.9)	.155	0.32	21 (26)	7 (9.3)	0.46
OMI	13 (52)	32 (41)	.359	0.23	41 (52)	29 (38)	0.28
Anteroseptal wall	5 (20)	14 (18)	.773	0.06	7 (8.8)	12 (16)	0.22
Inferior wall	9 (36)	17 (22)	.186	0.32	34 (43)	15 (20)	0.51
Posterior wall	4 (16)	12 (15)	>.99	0.02	17 (21)	11 (15)	0.17
Apical wall	0 (0)	1 (1.3)	>.99	0.16	0 (0)	1 (1.3)	0.16
History of PCI∗	11 (44)	37 (47)	.823	0.06	35 (44)	35 (47)	0.06
Emergency∗	0 (0)	2 (2.5)	>.99	0.23	0 (0)	0 (0)	0.00
Japan SCORE (%)	5.1 (3.2-8.5)	4.3 (2.8-6.8)	.055	0.40	4.6 (3.2-7.3)	4.5 (3.4-7.5)	0.09

Values are presented as the mean ± SD, median [interquartile range], or n (%). Weighted n values reflect the sum of weights after inverse probability of treatment weighting. *SMD*, Standardized mean difference; *COPD*, chronic obstructive pulmonary disease; *ESRD*, end-stage renal disease; *AVF*, arteriovenous fistula; *LDL-C*, low-density lipoprotein cholesterol; *LVDd*, left ventricular end-diastolic dimension; *LVDs*, left ventricular end-systolic dimension; *LVEF*, left ventricular ejection fraction; *LMT*, left main trunk; *LAD*, left anterior descending artery; *CTO*, chronic total occlusion; *RCA*, right coronary artery; *LCx*, left circumflex artery; *OMI*, old myocardial infarction; *PCI*, percutaneous coronary intervention; *Japan SCORE*, Japanese System for Cardiac Operative Risk Evaluation.

∗ Variables included as covariates in the propensity score model.

† Diabetes as the primary cause of ESRD at dialysis initiation (per nephrologist records), not diabetes mellitus as a comorbidity.

## Results

### Patient Characteristics and Intraoperative Findings

**Figure E1:**
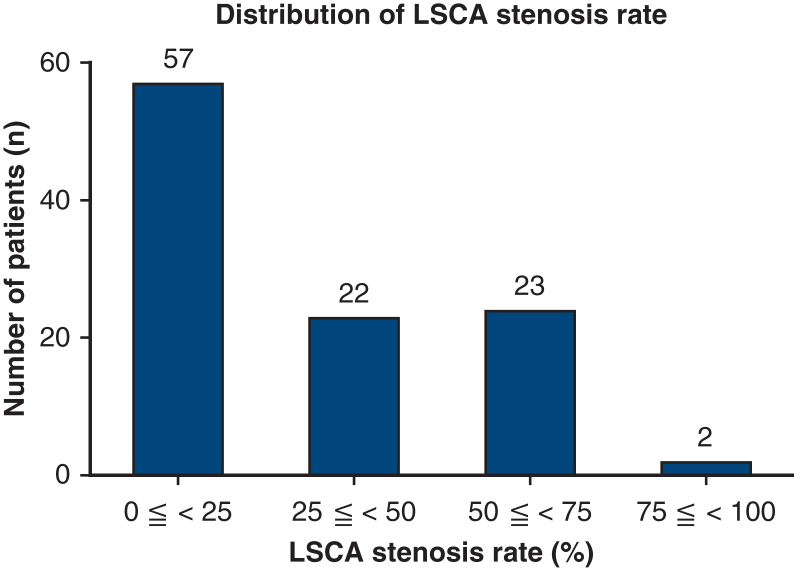
Preoperative left subclavian artery (LSCA) stenosis severity distribution (n = 104).

**Figure E2:**
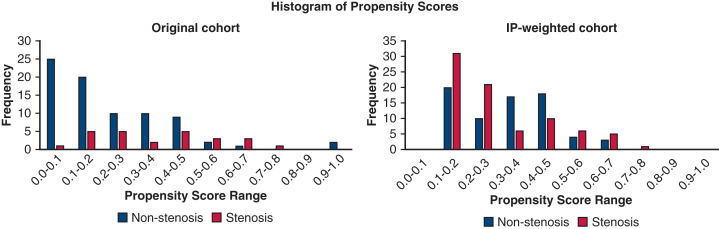
Propensity score distributions by group before and after inverse probability (IP) of treatment weighting.

**Figure E3:**
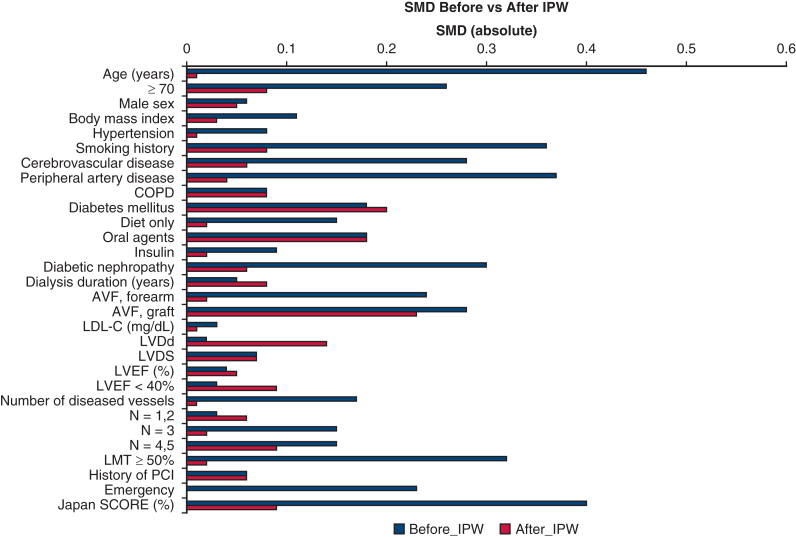
Covariate balance (absolute standardized mean difference [SMD]) before and after inverse probability of treatment weighting (IPTW) *COPD*, Chronic obstructive pulmonary disease; *AVF*, arteriovenous fistula; *LDL-C*, low-density lipoprotein cholesterol; *LVDd*, left ventricular end-diastolic dimension; *LVDs*, left ventricular end-systolic dimension; *LVEF*, left ventricular ejection fraction; *LMT*, left main trunk; *PCI*, percutaneous coronary intervention; *Japan SCORE*, Japanese System for Cardiac Operative Risk Evaluation.

**Figure E4:**
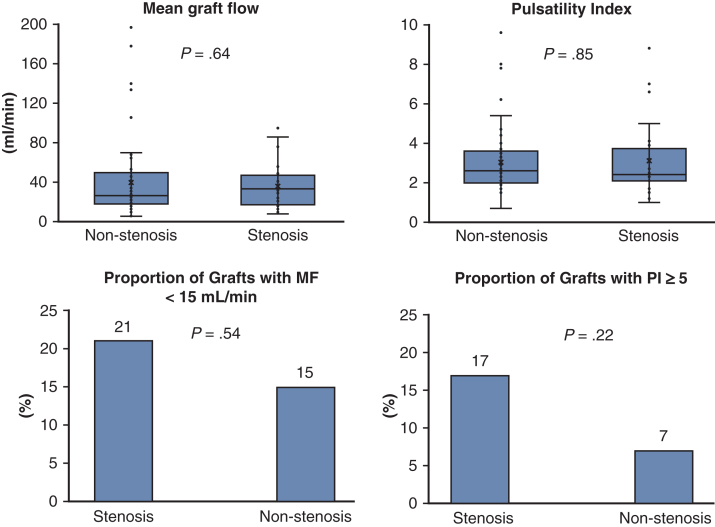
Intraoperative left internal thoracic artery-left anterior descending artery mean graft flow (MF) and pulsatility index (PI) by left subclavian artery stenosis status.

Of 104 hemodialysis patients, 25 had LSCA stenosis (≥50%) and 79 did not (Figure 1). Baseline characteristics were comparable after IPTW adjustment (Table 1 and Figure E1, Figure E2, Figure E3). Off-pump CABG was performed in 99% of cases. In the IPTW cohort, the LSCA stenosis group had a higher frequency of impaired LITA-LAD graft flow profiles (pulsatility index ≥5 and/or mean graft flow <15 mL/min) than the nonstenosis group (Table 2 and Figure E4). In-hospital mortality was 1.9% overall (2 out of 104), occurring only in the stenosis group (8.0% vs 0%). Other complications were similar, except for a higher rate of ventricular arrhythmia in the stenosis group (Table 3).

**Table 2:** Operative data

Variables	Original cohort (n = 104)				IPTW-weighted cohort (n = 154.6)		
	Stenosis (n = 25)	Nonstenosis (n = 79)	*P* value	SMD	Stenosis (n = 79.4)	Nonstenosis (n = 75.2)	SMD
OPCAB	25 (100)	78 (99)	>.99	0.16	79 (100)	75 (100)	0.00
Operative time (min)	258 ± 75	256 ± 57	.901	0.03	268 ± 83	252 ± 56	0.23
Preoperative IABP	0 (0)	2 (2.5)	1.00	0.23	0 (0)	0 (0)	0.00
Complete revascularization	23 (95)	75 (95)	.629	0.12	74 (93)	70 (93)	0.03
Other graft selection							
RITA	17 (68)	41 (52)	.174	0.33	57 (72)	38 (51)	0.45
Free RITA	0 (0)	3 (3.8)	>.99	0.28	0 (0)	3 (4.0)	0.29
GEA	2 (8.0)	17 (22)	.151	0.39	4 (5.1)	15 (21)	0.50
Free GEA	0 (0)	2 (2.5)	>.99	0.23	0 (0)	1 (1.3)	0.16
Saphenous vein	14 (56)	44 (56)	>.99	0.01	43 (54)	42 (56)	0.05
Composite graft (Y or I)	2 (8.0)	6 (7.6)	>.99	0.02	12 (15)	4 (5.3)	0.32
TTFM evaluation∗							
Mean flow (mL/min)	34 (17-47)	27 (18-50)	.643	0.12	29 (16-41)	25 (18-47)	0.19
Mean flow <15 mL/min	5 (21)	11 (15)	.536	0.14	18 (23)	9 (13)	0.27
PI	2.4 (2.1-3.7)	2.6 (2.0-3.6)	.846	0.04	2.4 (2.1-3.2)	2.7 (2.0-3.6)	0.03
PI ≥5	4 (17)	5 (7.0)	.224	0.30	10 (13)	4 (5.3)	0.25
DF (%)	73 ± 16	74 ± 8.2	.736	0.07	76 ± 13	75 ± 6.7	0.09
DF <50%	1 (4.2)	1 (1.4)	.443	0.17	2 (2.5)	0 (0)	0.23

Values are presented as mean ± SD, median (interquartile range), or n (%). Weighted n values reflect the sum of weights after inverse probability of treatment weighting (IPTW). *SMD*, Standardized mean difference; *OPCAB*, off-pump coronary artery bypass; *IABP*, intra-aortic balloon pump; *RITA*, right internal thoracic artery; *GEA*, gastroepiploic artery; *TTFM*, transit-time flow measurement; *PI*, pulsatility index; *DF*, diastolic filling fraction.

∗ TTFM values refer to the left internal thoracic artery-left anterior descending artery anastomosis.

**Table 3:** Early outcomes

Variables	Original cohort (n = 104)				IPTW-weighted cohort (n = 154.6)		
	Stenosis (n = 25)	Nonstenosis (n = 79)	*P* value	SMD	Stenosis (n = 79.4)	Nonstenosis (n = 75.2)	SMD
Postoperative stroke	0 (0)	1 (1.3)	>.99	0.16	0 (0)	1 (1.3)	0.16
Respiratory failure	2 (8.0)	1 (1.3)	.143	0.32	4 (5.1)	1 (1.3)	0.21
Arrhythmia	11 (44)	20 (25)	.085	0.40	30 (38)	23 (31)	0.14
POAF	8 (32)	18 (23)	.428	0.21	22 (28)	20 (27)	0.03
VT/VF	3 (12)	1 (1.3)	.042	0.44	8 (10)	1 (1.3)	0.38
DSWI	2 (8.0)	0 (0)	.056	0.29	11 (14)	0 (0)	0.56
In-hospital death	2 (8.0)	0 (0)	.056	0.29	4 (5.0)	0 (0)	0.32

Values are presented as n (%). *POAF*, Postoperative atrial fibrillation; *VT*, ventricular tachycardia; *VF*, ventricular fibrillation; *DSWI*, deep sternal wound infection.

### Follow-up and Graft Patency

Follow-up was 95.2% complete, with a mean of 4.6 ± 3.1 years (stenosis 3.2 ± 3.0 years vs nonstenosis 5.0 ± 3.1 years; *P* = .011). Early postoperative imaging (≤3 months; n = 32) showed 100% graft patency. Late imaging (>3 months; mean, 3.3 ± 2.4 years) also suggested high LITA-LAD patency (stenosis 7 out of 7, nonstenosis 31 out of 33); however, these findings are descriptive due to selection bias (available in only 28% of the stenosis group) (Table E2). CT-based LSCA assessment showed excellent reproducibility (interobserver intraclass correlation coefficient: 0.90; intraobserver intraclass correlation coefficient: 0.92).

### Primary and Secondary Outcomes

**Figure 2:**
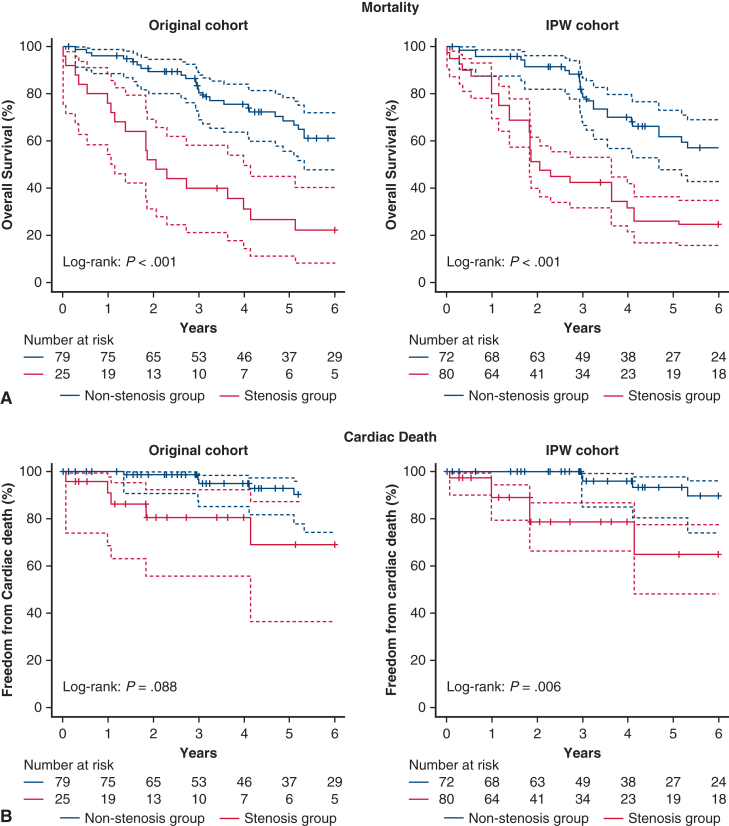
Study outcomes Kaplan-Meier survival curves for overall survival (A) and cardiac-death-free survival (B) in the original and inverse probability of treatment weighting-adjusted cohorts. Dashed lines indicate 95% CI. *P* values and numbers at risk are shown.

During follow-up, 59 deaths occurred (Table E1). The 5-year survival was significantly lower in the LSCA stenosis group in both the original (27% vs 69%; *P* < .001) and IPTW cohorts (26% vs 62%; *P* < .001) (Figure 2). In the doubly robust Cox model, LSCA stenosis independently predicted higher all-cause mortality (hazard ratio, 2.62; 95% CI, 1.25-5.49) (Table E3). Cardiac death was significantly higher in the IPTW cohort (*P* = .006) but not in the original cohort (*P* = .088), whereas MACE showed no significant difference (Table 4). Multivariable Cox regression confirmed LSCA stenosis as an independent predictor of all-cause mortality (hazard ratio, 3.14; 95% CI, 1.66-5.93) (Table 5).

**Table 4:** Long-term clinical outcomes

Outcome	Original cohort (n = 104)				IPTW-weighted cohort (n = 154.6)			
	Stenosis (n = 25)	Nonstenosis (n = 79)	Hazard ratio (95% CI)	*P* value	Stenosis (n = 79.4)	Nonstenosis (n = 75.2)	Hazard ratio (95% CI)	*P* value
Primary outcome								
Overall survival	0.27 (0.11-0.45)	0.69 (0.56-0.78)	2.66 (1.54-4.60)	<.001	0.26 (0.17-0.36)	0.62 (0.48-0.73)	2.09 (1.39-3.15)	<.001
Secondary outcome								
Cardiac-death-free survival	0.69 (0.36-0.87)	0.15 (0.06-0.24)	2.56 (0.87-7.56)	.088	0.65 (0.48-0.78)	0.93 (0.81-0.98)	3.68 (1.46-9.30)	.006
MACE	0.29 (0.11-0.46)	0.69 (0.56-0.78)	1.42 (0.63-3.18)	.400	–∗	–∗	1.30 (0.54-3.11)	.553

Values represent 5-y estimates (survival for all-cause and cardiac death; cumulative incidence for major adverse cardiac event [MACE]) with 95% CI. Overall survival and cardiac-death–free survival were estimated using the Kaplan-Meier method; hazard ratios were estimated using Cox proportional hazards models. MACE was assessed using competing-risk methods (Gray test and Fine-Gray subdistribution hazard models), treating noncardiac death as a competing event. Inverse probability of treatment weighting was applied for weighted analyses; weighted n reflects the sum of weights. *MACE*, Major adverse coronary event.

∗ For MACE, group-specific estimates were not reported due to numerical instability in the inverse probability of treatment-weighted analysis.

**Table 5:** Risk factor analysis for all-cause mortality (original cohort)

Variables	Multivariable analysis	
	Hazard ratio (95% CI)	*P* value
Age	1.05 (1.01-1.08)	.004
Male sex	0.75 (0.35-1.59)	.452
Cerebrovascular disease	1.11 (0.54-2.30)	.769
Peripheral artery disease	1.76 (0.97-3.21)	.064
LSCA stenosis ≥50%	3.14 (1.66-5.93)	<.001
COPD	0.35 (0.07-1.75)	.200
Primary cause of ESRD: diabetes∗	0.94 (0.48-1.83)	.851
Dialysis duration	0.97 (0.91-1.04)	.403
AVF, forearm	1.00 (0.37-2.67)	.994
LVEF	0.98 (0.96-1.00)	.113
LMT disease ≥50%	0.58 (0.29-1.16)	.129
History of PCI	0.98 (0.54-1.78)	.937

Hazard ratios were estimated using Cox proportional hazards models; *P* values were derived from Wald tests. Continuous covariates were modeled per unit increase as indicated (age, per 1 year; dialysis duration, per 1 year; LVEF, per 1%). *LSCA*, Left subclavian artery; *COPD*, chronic obstructive pulmonary disease; *ESRD*, end-stage renal disease; *AVF*, arteriovenous fistula; *LVEF*, left ventricular ejection fraction; *LMT*, left main trunk; *PCI*, percutaneous coronary intervention.

∗ Diabetes recorded as the primary cause of ESRD at dialysis initiation.

## Discussion

Moderate LSCA stenosis is not a benign finding in patients requiring hemodialysis undergoing LITA-LAD CABG. Even when asymptomatic, moderate (≥50%) stenosis was independently associated with increased long-term mortality and cardiac death. These findings indicate that even modest inflow restriction can have clinically significant consequences in this high-risk population.

### Mechanistic Considerations

Because the LITA originates from the SCA, its perfusion pressure is lower than that of the ascending aorta.^17^ Even moderate LSCA stenosis further reduces diastolic inflow pressure, compromising graft hemodynamics. Prior studies show that ≥50% subclavian narrowing causes vertebral artery flow reversal in more than 90% of patients,^18^^,^^19^ disturbing hemodynamics even when clinically silent. Consistently, the stenosis group exhibited more impaired LITA-LAD flow profiles and frequent ventricular arrhythmias, including episodes during hemodialysis. Inadequate LITA-LAD perfusion likely contributes to both perioperative and long-term adverse outcomes. Increased AVF flow during dialysis can precipitate CSS. In patients with moderate stenosis, dialysis-related stress may transiently or persistently reduce myocardial perfusion in the LAD territory. Furthermore, progressive subclavian disease can amplify long-term ischemic risk over time.

### Context Within the Literature

Previous reports often suggested ipsilateral ITA grafting is safe,^6^, ^7^, ^8^, ^9^, ^10^ largely because LSCA stenosis was not systematically evaluated. Although clinical surrogates like interarm blood pressure differences are used,^9^ they cannot reliably detect subclinical disease. A recent meta-analysis identified subclavian stenosis—rather than AVF laterality—as the key determinant of CSSS.^4^ Our results extend this by establishing moderate LSCA stenosis as a measurable, modifiable risk factor. These data challenge the assumption that in situ LITA-LAD grafts are universally safe in patients receiving hemodialysis and mandate routine preoperative subclavian assessment.

### Clinical Implications

The present findings have direct implications for surgical strategy. Routine preoperative subclavian imaging should be considered in patients requiring hemodialysis undergoing CABG. When moderate LSCA stenosis is detected, individualized inflow management is crucial.

Beyond preoperative endovascular intervention, converting the LITA to a free graft with aortic inflow represents a rational alternative that ensures adequate perfusion pressure independent of the subclavian circulation. This is particularly beneficial with left-arm AVFs, where flow competition can further diminish graft inflow. Other options, such as using a free right ITA or GEA, may also be appropriate. Moreover, postoperative vascular surveillance should be implemented to detect progressive inflow compromise. Collectively, these insights support incorporating subclavian assessment into preoperative planning: preoperative subclavian evaluation and flexible inflow selection must become integral to surgical planning in this high-risk population.

### Study Limitations

This study has several limitations. First, the sample size was limited by stringent inclusion criteria. Although IPTW mitigated baseline differences, residual confounding remains; specifically, LSCA stenosis may reflect advanced systemic atherosclerosis as well as impaired subclavian inflow physiology, rather than being the sole cause of adverse outcomes. Second, although a priori selection based on LSCA status was unlikely, selection bias regarding preoperative CT implementation cannot be entirely ruled out. Third, postoperative imaging and functional AVF assessments were not systematically performed. Fourth, although post-CABG subclavian interventions were not prespecified, 1 patient required intervention for symptomatic CSSS, highlighting the clinical relevance. Fifth, vascular access revisions during follow-up were not systematically captured. Finally, the long study period (2009-2022) encompassed advances in perioperative care and pharmacotherapy. Future multicenter, prospective studies using functional imaging—such as CT-derived fractional flow reserve—are warranted to validate these findings and elucidate the interplay between dialysis-related hemodynamics and graft outcomes.

## Conclusions

Moderate preoperative LSCA stenosis independently predicts increased long-term mortality and cardiac death after LITA-LAD CABG in patients requiring hemodialysis. These findings emphasize the need for routine subclavian evaluation and tailored graft strategies, including aortic inflow conversion, to optimize outcomes in this high-risk population.

### Declaration of Generative AI and AI-Assisted Technologies in the Writing Process

During the preparation of this work, the authors used ChatGPT (OpenAI) to improve grammar and wording. After using this tool/service, the authors reviewed and edited the content as needed and take full responsibility for the content of the publication.

## Conflict of Interest Statement

The authors reported no conflicts of interest.

The *Journal* policy requires editors and reviewers to disclose conflicts of interest and to decline handling or reviewing manuscripts for which they may have a conflict of interest. The editors and reviewers of this article have no conflicts of interest.

## Figures and Tables

**Table E1:** Breakdown of adjudicated causes of death

Variables	Stenosis (n = 25)	Nonstenosis (n = 79)	Total (N = 104)
All-cause death	20 (80)	39 (49)	59 (57)
Cardiac death	5 (20)	11 (14)	16 (15)
Non-cardiac death	13 (52)	18 (23)	31 (30)
Undetermined	2 (8)	10 (14)	12 (12)

Values are presented as n (%). Deaths with insufficient information were classified as “undetermined” to ensure a conservative assessment of cardiac death. In a descriptive sensitivity analysis where all undetermined deaths were hypothetically reclassified as cardiac deaths, the cardiac death rates were 7 out of 25 (28%) in the left subclavian artery stenosis group and 21 out of 79 (27%) in the nonstenosis group.

**Table E2:** Late postoperative graft patency among patients with available imaging

Variables	Stenosis	Nonstenosis	Total
Late graft imaging available	7/25 (28)	33/79 (42)	40/104 (38)
LITA patency	7/7 (100)	31/33 (94)	38/40 (95)
RITA patency	6/6 (100)	17/18 (94)	23/24 (96)
GEA patency	1/1 (100)	8/8 (100)	9/9 (100)
SVG patency	1/2 (50)	15/20 (75)	16/22 (73)
Time from CABG to late imaging (y)	2.9 ± 2.3	3.4 ± 2.4	3.3 ± 2.4

Values are presented as n/N (%) or mean ± SD. Late imaging was defined as imaging performed more than 3 months after CABG. The first row is patient-level; subsequent rows are conduit-level. Patency was assessed at the conduit level (LITA, RITA, SVG, and GEA); sequential grafts were counted as a single conduit, and patency was not adjudicated for each individual distal anastomosis. Denominators represent the number of conduits assessable by late imaging, rather than the total number of implanted conduits. One LITA-left anterior descending artery graft exhibited a string sign without objective evidence of ischemia and was classified as patent. Imaging modalities primarily included coronary computed tomography angiography and selected invasive coronary angiography. These results are descriptive because postoperative imaging was not performed systematically. *LITA*, Left internal thoracic artery; *RITA*, right internal thoracic artery; *GEA*, gastroepiploic artery; *SVG*, saphenous vein graft; *CABG*, coronary artery bypass grafting.

**Table E3:** Doubly robust sensitivity analysis for all-cause mortality

Variables	Hazard ratio	95% CI	*P* value
LSCA stenosis	2.62	1.25-5.49	.010
MGF <15 mL/min	1.71	0.79-3.67	.171
Diabetes mellitus	0.84	0.38-1.85	.669
RITA use	0.54	0.29-1.01	.053
Prior MI	0.78	0.30-2.04	.619

Weighted Cox proportional hazards models were fitted using inverse probability of treatment weights and additionally adjusted for covariates with residual imbalance after weighting (standardized mean difference ≥0.20). Robust (sandwich) SEs were used to account for variance inflation induced by weighting. *LSCA*, Left subclavian artery; *MGF*, mean graft flow; *RITA*, right internal thoracic artery; *MI*, myocardial infarction.
